# Neurologic Involvement in Scleroderma en Coup de Sabre

**DOI:** 10.1155/2012/719685

**Published:** 2012-01-27

**Authors:** Tiago Nardi Amaral, João Francisco Marques Neto, Aline Tamires Lapa, Fernando Augusto Peres, Caio Rodrigues Guirau, Simone Appenzeller

**Affiliations:** ^1^Rheumatology Division, Faculty of Medical Science, State University of Campinas, 13083-970 Campinas, SP, Brazil; ^2^Postgraduate Program in Child and Adolescent Health, Faculty of Medical Science, State University of Campinas, 13083-970 Campinas (UNICAMP), Brazil; ^3^Rheumatology Lab, Faculty of Medical Science, State University of Campinas, 13083-970 Campinas, SP, Brazil

## Abstract

Localized scleroderma is a rare disease, characterized by sclerotic lesions. A variety of presentations have been described, with different clinical characteristics and specific prognosis. In scleroderma en coup de sabre (LScs) the atrophic lesion in frontoparietal area is the disease hallmark. Skin and subcutaneous are the mainly affected tissues, but case reports of muscle, cartilage, and bone involvement are frequent. These cases pose a difficult differential diagnosis with Parry-Romberg syndrome. Once considered an exclusive cutaneous disorder, the neurologic involvement present in LScs has been described in several case reports. Seizures are most frequently observed, but focal neurologic deficits, movement disorders, trigeminal neuralgia, and mimics of hemiplegic migraines have been reported. Computed tomography and magnetic resonance imaging have aided the characterization of central nervous system lesions, and cerebral angiograms have pointed to vasculitis as a part of disease pathogenesis. In this paper we describe the clinical and radiologic aspects of neurologic involvement in LScs.

## 1. Introduction

Scleroderma is a rare disease of unknown etiology, characterized by thickening and hardening of skin resulting from increased collagen production. The term includes a variety of diseases, from localized scleroderma (LS) to systemic sclerosis. LS is traditionally considered to be limited to skin, subcutaneous tissue, underlying bone, and, in craniofacial subtype, nervous system involvement [1]. Recent studies, however, have described malaise, fatigue, arthralgia, and myalgia in morphea. Moreover, rheumatologic, ophthalmologic and neurologic symptoms and signs have been described in up to 20% of the patients with LS. Based on these findings LS ought to be differentiated from systemic sclerosis by the absence of sclerodactylya, Raynaud's phenomenon, and capillaroscopic abnormalities [1].

 LS incidence ranges from 0.4 to 2.7 per 100,000 people [2]. Although present in all races, the prevalence among Caucasians is increased, summing up 72 to 82% of the patients [2]. Females are primarily affected [1], and a similar distribution between children and adults occurs [1, 3]. Disease incidence peaks in the fifth decade of life in adults, whereas 90% of children are diagnosed between 2 and 14 years of age [1, 3–5].

 Linear scleroderma en coup de sabre (LCsc) is a rare subset of LS. The typical presentation affects frontoparietal region, and the mean age of onset is around 13 years old [1]. In this paper, clinical presentation of LScs and its neurological involvement are described.

## 2. Pathogenesis

Skin pathogenesis seems to be similar between LScs, LS, and systemic sclerosis, although not fully understood [1, 6–8]. Clinical and pathological data support the hypothesis that vasculature is the primary target in LS [6, 7, 9]. Early skin biopsies revealed damaged endothelial cells preceding the development of fibrosis by months to years. Increased vascular permeability is associated with mononuclear cell infiltration, leading to perivascular inflammatory cell infiltrates, vascular intimal thickening, and vessel narrowing [8]. Gradually, the vessels lose their elasticity; media and adventitia become fibrotic and more prone to small-artery occlusion. The latter is further exacerbated by thrombotic events driven by platelets activation, resulting in fibrosis and end-organ damage [8].

The inciting event for microvascular damage remains unknown. Preceding trauma has been observed as initial event in pediatric population [10, 11]. Previous infection, particularly due to *Borrelia burgdorferi*, has been implicated in Europe and Japan, but not confirmed in the United States [12, 13]. Genetics participation in pathogenesis appears to be relatively weak, since only a 4.7% concordance between twins has been observed [14] and family studies revealed only 1.6% frequency among first-degree relatives [8, 15]. However, several groups have identified polymorphisms in potential candidate genes involved in immune regulation, such as BANK1, C8orfl3-BLK, IL-23R, IRF5, STAT4, TBX21, and TNFSF4, which may underlie the pathogenesis of systemic sclerosis [8, 16]. Intriguingly, many of these polymorphisms are shared with other rheumatic diseases, such as systemic lupus erythematosus.

More than 1800 genes are differentially expressed in scleroderma skin compared to healthy controls; however analysis of visually unaffected skin reveals a similar gene expression as diseased skin [17, 18]. Altered gene expression is mapped to fibroblasts, endothelial, epithelial, smooth muscle, T, and B cells [8]. Recently, Gardner et al. have found significant gene-expression signature in systemic sclerosis patients, which has been mapped to TGF-*β* and WNT signaling pathways, the production of extracellular matrix proteins and CCN family proteins [18].

Pathogenesis of CNS involvement in LScs is due to perivascular infiltrate and vasculitis [9, 19, 20]; however biopsy is not routinely done and histological findings are available only for patients with severe neurological findings. Gliosis, suggesting chronic inflammatory process, leptomeningeal band-like sclerosis, and thickened blood vessels' walls, as well as intraparenchymal calcification, have also been described in the few available studies [9, 19, 20].

To sum up, available data suggests a complex pathogenesis of scleroderma, in which blood vessels, the immune system, and extracellular matrix are affected and may contribute to the development of the disease.

## 3. Clinical Presentation

Ivory-colored, sclerotic lesions, with violaceous borders, characterize LS. Number and distribution of lesions vary, as well as their extent. These characteristics and tissue involvement (dermis, subcutaneous tissue, fascia, and muscle) determine the localized scleroderma classification (Table 1) [1, 21].

LScs presents in a band-like fashion on the frontoparietal scalp and forehead. Alopecia is common and many times is the patient's main concern. Skin lesions may extend to the nose, cheek, chin, and neck [8, 22, 23] and usually have an active stage lasting 2–5 years [24, 25]. Muscle, cartilage, and bone lesions incur in facial atrophy: in this scenario, Parry-Romberg syndrome (PRS) must be considered a differential diagnosis. Up to 28% of patients having LScs manifests PRS features, such as a unilateral slowly progressive atrophy of the face. PRS commonly affects dermatomes of trigeminal nerve. Skin, soft tissue muscles, and underlying bone structures are involved [26]. Skin hyperpigmentation and discoloration and hairless patches can be present. Many authors postulate that LScs and PRS are clinical variants of the same disease. Arguments for PRS inclusion on the spectrum of LS disorders are compelling. LScs and PRS coexist in 20–37% of the patients with LScs diagnosis, and both conditions have similar age of onset and disease course [27]. Furthermore, dermatologic findings in PRS are sometimes indistinguishable from those of LS [27]. However, some authors still consider them as different entities, since PRS does not always have skin thickening [28–30] and the hemifacial atrophy occurring in PRS is usually more prominent [25] (Table 2).

 The diagnosis is clinical and based on characteristic cutaneous and soft tissue findings [6, 24, 25]. Currently no diagnostic laboratory tests exist. Nonetheless, 37–50% of the patients may present a positive ANA test (homogenous or speckled patterns) [2, 6, 24], as well as anti-single-stranded-DNA antibodies [5, 31, 32]. Antinucleosome antibodies, soluble interleukine-2 receptor, and, recently, antiagalactosyl immunoglobulin G antibodies have been reported in LS [33–35]. In some patients, autoantibody may be present even before the disease manifestation and patients with Scl-70, anticentromere, Ro/La, or U1RNP antibodies should be followed closely, as systemic disease might ensue [6].

## 4. Neurologic Involvement

LScs has been associated with a variety of neurologic abnormalities and typically is preceded by the development of cutaneous disease by months to years [8, 31, 36, 37]. Nervous system involvement is usually not correlated to skin activity and may present years after the disease initial symptomatology [37]. In 16% of cases, neurologic symptoms predate the cutaneous manifestations [27].

Neurological symptoms and signs in LScs are protean and include epilepsy [8, 38, 39], headache [27, 40], focal neurologic deficits, and movement disorders [27, 31, 36, 41, 42], as well as neuropsychiatric symptom and intellectual deterioration [43–45].

### 4.1. Epilepsy

Epilepsy is a frequently reported manifestation in LScs. An analysis of 54 patients with LScs or PRS has revealed a prevalence of 73% of seizures, 33% of them refractory to antiepileptic medications [27]. Complex partial seizures have been reported most frequently, followed by tonic-clonic, absence seizures, as well as status epilepticus [9, 38, 39]. Electroencephalography analyses show abnormalities in the majority of patients. Some authors advocate that brain lesions of LS are more epileptogenic than those of other autoimmune disorders [27].

### 4.2. Focal Neurologic Deficits and Movement Disorders

Focal neurologic deficits and movement disorders secondary to brain lesions have also been described, but seem to be relatively uncommon [27, 36, 41]. In an analysis of 54 patients, focal neurological deficits were described in 11% of patients at presentation and in 35% of patients overall [27]. While facial palsy and extraocular movement disorder may be due to cutaneous involvement, trigeminal neuralgia [31] and masticatory spasms [42] are considered primary neurologic involvement.

### 4.3. Other Neurological Findings

Around 35% of LScs patients refer headache, which is usually associated with other neurologic complaints [27]. Few studies have investigated headache subtype, but migraines and mimics of hemiplegic migraine seem to be more prevalent [27, 40].

Neuropsychiatric symptoms have been described in 15% of patients, including behavioral changes and progressive intellectual deterioration with [43–45] or without seizures [46].

## 5. Neuroimaging

Computed tomography (CT) and magnetic resonance (MRI) studies have shown central nervous system abnormalities in LScs patients. Neurologic findings are more frequently ispilateral to the skin lesions, but contralateral involvement has been described [19, 36]. Neurologic symptoms should not be used as a predictor for MRI abnormalities because neurologic lesions have been discovered in asymptomatic patients [30, 47]. Moreover, symptomatic patients were sometimes proven to have normal radiologic exams.

Outer diploe thinning, cerebral atrophy, white matter lesions, focal subcortical calcifications, and meningocortical alterations have been described [30, 35]. Intraparenchymal calcifications involving basal ganglia, thalami, and dentate nuclei are the most common brain lesion in LScs patients [30, 47, 48]. Characteristically, the calcifications are ipsilateral [46, 49], but contralateral involvement may occur [36, 50].

MRI usually exhibits T2 hyperintensities, mostly in subcortical white matter, but also in corpus callosum, deep grey nuclei, and brain stem [29, 30, 40, 46, 47, 51, 52].

Cerebral atrophy is generally subtle, characterized by blurring of the gray-white interface, cortical thickening, and abnormal gyral pattern [30]. Atrophy is usually focal but widespread lesions involving an entire cerebral hemisphere have been described [30, 46, 52]. Hippocampal atrophy is unusual, but has been reported [28, 35]. Infratentorial lesions and cerebellar hemiatrophy have been observed in patients presenting more severe neurological symptoms [27].

Cerebral angiograms and magnetic resonance angiograms studies showed vascular involvement suggestive of vasculitis. Reports of cerebral aneurysms and other vascular malformations, as brain cavernomas [48, 53, 54], exist and could represent late sequelae of vasculitic process.

## 6. Treatment

At this moment, no randomized controlled trials exist for LScs. In a retrospective study of LScs and/or PRS patients conducted at a tertiary care center, antimalarials, methotrexate, topical and oral steroids, and tetracycline were used for cutaneous disease, but no definite conclusions could be drawn due to the small sample size and the absence of a control group [55]. D-penicillamine, methylprednisolone, mycophenolate mofetil, and methotrexate might be considered in the treatment of neurologic involvement of LScs [9]. In reported cases, association of methotrexate or mycophenolate mofetil and steroids appeared to have impact in controlling intractable seizures and stabilizing central nervous system damage [27, 40, 44, 48, 56].

## 7. Conclusion

Once believed to exclusively involve skin, subcutaneous tissue, and bone, LS has been associated to systemic symptoms. Rheumatologic, ophthalmologic, and neurologic manifestations seem to be present in around 20% of the patients, and, in those with LScs, nervous system disorders are the most prevalent extracutaneous presentation.

Neurologic damage in LScs is frequent and independent of clinical signs and symptoms. Radiologic findings and pathologic studies point towards a neurovasculitic hypothesis. The investigations of choice are CT, to detect skull abnormalities, and MRI, to identify underlying brain lesions. Neuroimaging studies should be considered in all LScs patients at the time of the diagnosis. Longitudinal studies should be done to identify progression, even in asymptomatic patients.

## Figures and Tables

**Table 1:** Localized scleroderma classification.

Classification	Subtypes	Characteristic lesions	Tissues involved	Main Site
Circumscribed	Superficial variant	Oval lesions	Limited to epidermis and dermis	Trunk
morphea	Deep variant	Oval lesions	Deep indurations. Dermis and subcutaneous tissue involved. Variable muscle and fascia involvement	Trunk

Linear morphea	Trunk/limb variant	Linear indurations	Dermis and subcutaneous tissue (may involve muscle and bone)	Trunk/limb
(linear scleroderma)	Head variant (en coup de sabre)	Linear indurations	Dermis of the frontoparietal area (may involve muscle, bone, and central nervous system)	Face and scalp
	Parry-Romberg syndrome		Dermis, subcutaneous tissue, muscle, cartilage, and bone	Unilateral face

Generalized morphea		Four or more indurated plaques >3 cm each	Usually limited to the dermis and rarely involves subcutaneous tissue	Diffuse (no face and hand)

Pansclerotic morphea		Circumferential involvement	Epidermis, dermis, subcutaneous tissue, muscle and bone	Limbs

Mixed variant morphea			Combination of 2 or more previous subtypes	

**Table 2:** Clinical aspects of linear scleroderma en coup de sabre (LScs) and Parry-Romberg syndrome (PRS).

	LScs	PRS
Skin	Induration and thickening	Not affected

Initial site	Forehead and scalp	Cheek and nose

Spreading pattern	Usually does not spread below the forehead	Usually affects lower face
	Occasionally affects nose, cheek, chin, and neck	Usually restricted to one side
	Occasionally bilateral	

Systemic involvement	Yes	No

Intracranial involvement	Yes	Yes
